# Prevalence of mental disorders among adolescents in German youth welfare institutions

**DOI:** 10.1186/1753-2000-2-2

**Published:** 2008-01-28

**Authors:** Marc Schmid, Lutz Goldbeck, Jakob Nuetzel, Joerg M Fegert

**Affiliations:** 1 Department of Child and Adolescent Psychiatry/Psychotherapy, University Basel, Switzerland; 2 Department of Child and Adolescent Psychiatry/Psychotherapy, University Hospital Ulm, Germany; 3 Department of Child and Adolescent Psychiatry/Psychotherapy, Centrum for Psychiatry the Weissenau Ravensburg, Germany

## Abstract

**Objective:**

Multiple psycho-social risk factors are common in children and adolescents in youth welfare, especially in residential care. In this survey study we assessed the prevalence of behavioral, emotional symptoms and mental disorders in a German residential care population.

**Methods:**

20 residential care institutions including 689 children and adolescents (age 4 – 18 years; mean 14.4; SD = 2.9) participated. A two-step design was performed. First, the children and adolescents and their residential caregivers answered a standard symptom checklist (CBCL/YSR). For those participants scoring more than one standard deviation above the mean of their German population reference group, a standardized clinical examination was performed to specify an ICD-10 diagnosis.

**Results:**

The study population reached high average scores in almost all scales and subscales of the CBCL and YSR (mean CBCL total score T = 64.3, SD = 9.7, Median = 66.0). The prevalence of mental disorders according to the diagnostic criteria of ICD-10 was 59.9%, with a predominance of externalizing and disruptive disorders. High rates of co-morbidity were observed.

**Conclusion:**

Children and adolescents in youth welfare and residential care are a neglected high risk population. Providing adequate psychiatric diagnosis and multimodal treatment for this group is necessary.

## Introduction

Multiple risk factors such as poverty, broken homes, neglect, sexual and physical abuse, discontinuous relationships, and genetic factors have an impact on the mental health of children and adolescents in residential or foster care [1-5]. These children and adolescents have a very high risk for the development of a chronic mental disorder with subsequent impairment of their psychosocial functioning, for example school failure, unemployment or a criminal career [6,7]. In follow up studies 19% of the children moved through three or more different foster families or institutions [8,9].

Moving placements and repeated breakdowns of supporting youth welfare measures may worsen the prognosis because of the detrimental effects of the loss of attachment figures on the psychosocial development. So far there are only little data about the mental health status of these children and adolescents, because epidemiologic studies often restrict their research on children and adolescents living with their biological parents [10]. Survey studies on children in group homes are scarce, and the results on the prevalence of mental disorders in this population differ within a wide range.

Table 1 gives an overview over the prevalence rates found in different studies, most of them have been conducted in anglo-american countries. The review of the literature demonstrates sufficient evidence for the fact that mental disorders are significantly more frequent in residential care populations than in the general population [11]. Variations in prevalence estimates may be due to methodical and sampling effects since different diagnostic measures and criteria have been applied. Unknown selection biases may have distorted the prevalence rates and most of the studies did not control their drop-out rate. Moreover it is unknown whether the study samples represent typical populations of children and adolescents in the respective child welfare systems. Because the child and youth welfare services are different in every country, it is difficult to generalize the findings from one country to another. The threshold to place children and adolescents outside their biological families may differ between countries according to different legal and cultural backgrounds. There is a lack of mental health surveys using specific diagnostic criteria in German residential care populations. Graf et al. [12] reported an 80% prevalence of mental disorders in a study of 103 children and adolescents in German group homes, but this study was based only on a general clinical judgment without specifying diagnostic criteria and has not been replicated yet.

**Table 1:** Overview of prevalence rates in different studies

Study	Sample	Sample size	Prevalence	Instruments	ICD-10 diagnoses
McIntyre and Keesler 1986 [32]	foster care	N = 158	48.7%	CBCL (1)	No
McCann et al. 1996 [23]	foster & residential care	N = 103 n = 38 in residential care	96% in residential care 57%in foster care	CBCL Kiddie-sads (4)	Yes
Minnis et al. 2001 [33]	foster care	N = 182	60%	SDQ (2)	No
Hukkanen R. et al. 1999 [25]	residential care	N = 91	59%	CBCL & TRF	No
Dimigen et al. 1999 [24]	residential and foster	N = 70	30–50% in the different subscales	Devereux Scales of mental disorders (3)	No
Graf et al. 2002 [12]	residential care	N = 103	80%	Clinical Diagnoses	Yes
Meltzer et al. 2003 [2] Ford et al. 2007 [3]	foster & residential care	Total 1039 (N = 168 residential care)	Total 45–49% 68% (residential care)	SDQ clinical interview	Yes
Burns et al. 2004 [1]	foster & residential care	N = 3803	88,6% in residential care 63,1% in foster care	CBCL	No
Blower et al. 2004 [26]	foster & residential care	N = 48	44% in residential care	CBCL & Kiddie sads	Yes
Mount et al. 2004 [34]	foster & residential care	N = 50	70%	SDQ	No

(1) Child Behavior Checklist (CBCL) [15],

(2) Strength and Difficulties Questionnaire (SDQ) [31],

(3) Devereux Scales of mental disorders [35], Kiddie-Sads [30]

In the present multi-site study, we wanted to estimate the prevalence of mental disorders in a German residential care population by a psychometric and clinical examination. To avoid selection biases, one demand on this study was that a total population of children and adolescents living in the participating institutions should be included.

## Methods

### Recruitment strategy and sample description

From an official inventory edited by the state Baden-Wuerttemberg all youth welfare institutions offering group homes in the vicinity of the study centre were invited to an information event. 24 institutions followed the invitation; three others did not attend but replied that they were interested. 20 out of the 27 institutions with 689 children and adolescents ended up participating in the study. Finally half of all 1227 officially registered residential care children in eastern Baden-Wuerttemberg [13] were included in this study. Because of this good resonance systematic selective distortion of the institution sample is unlikely but could not be controlled and excluded scientifically. Seven child welfare institutions were not able to participate because of imminent structural changes within the institution, employee turnover, or high workloads not allowing data collection. The sample comprises institutions of various sizes. The smallest institution cared for six children and adolescents, the largest for 106. The large institutions are subdivided in smaller residential buildings and groups. 12 institutions provided a special school, and 14 had integrated a psychological service. The mean group size in our sample was 8.4 children, in average looked after by 2.6 educators. Compared with the characteristics of all registered institutions, our sample represents a good cross section of the whole residential care situation in Germany compared with information's from the Youth Welfare Services of the states Bayern [14] and Baden-Wuerttemberg [13]. Children and adolescents in residential care within the age range between 4 and 18 years were included. Some adolescents reached their 19^th ^birthday during the time span between screening and clinical examination.

After building up the co-operation with the institutions, informed consent of the person who holds custody and assent of the children and adolescents to participate in the study were acquired, following the principles of the local ethics committee. If no informed consent could be obtained, for example due to a lack of personal contact or engagement, the custodian in charge within the institution collected the screening data and passed it over to the study centre in an anonymous way, in order to control for a possible selection bias. This procedure was approved by the local ethics committee.

557 children and adolescents (397 male, 160 female) with a mean age of 14.4 years (SD = 3.0, range 4–19 years, median = 15.0) participated in the mental health screening. In addition anonymous caregiver-reports were collected for 132 children and adolescents. In average, the children and adolescents have been living in their institutions for 2.17 (SD = 2.3) years. The vocational status of their parents indicated low socio-economic status of all of the participants' families. 81% of the biological parents were separated at the time of assessment or had never lived together. 45.2% of the children attended special schools.

### Study design and instruments

**Figure 1:**
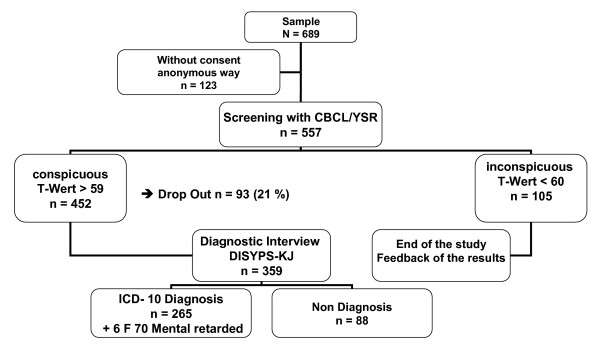
Design of the study and distribution of individuals.

A two step design was performed (compare Figure 1). First the residential care educators completed the *Child Behavior Checklist CBCL 4–18 *[15].

Children of age 11 or older filled in the *Youth Self Report YSR *[16]. The CBCL and the YSR are internationally widespread screening instruments for the assessment of psychopathology of children and adolescents. The CBCL contains 113 items/symptoms of psychopathology, grouped into eight subscales and three global scales. At level of global scores, externalizing and internalizing symptoms can be differentiated. Reliability and validity of the YSR/CBCL has been established repeatedly [17]. The internal consistency scores of the German version determined with Cronbach's alpha are between .81 and .92 for the three global scales [18]. The results in this sample are comparable with the findings of Doepfner et al. [18]. For the Youth Self Report global Scales Cronbach's alpha from .86 to .93 and for the Child Behavior Checklist global scales Cronbach's alpha between .85 and .94 could be calculated.

As a global measure of psychosocial functioning, the residential care educator also completed the *Children's Global Assessment Scale CGAS *[19,20]. The CGAS discriminates between ten different levels of global social functioning. The test-retest reliability of the CGAS is r = .85.

A CBCL/YSR total-score of 60 T-points discriminates best between children with and without mental disorders [21]. Therefore only those individuals who scored more than 59 T-points in the YSR and/or in the CBCL global score were subsequently (within 2 to 12 weeks after screening procedure) interviewed to confirm or exclude an ICD-10 diagnosis. Those disorders which are known to have the highest base rates in a general child and adolescent population (anxiety, depression, conduct disorder, and ADHD) were diagnosed using the *Diagnostic System for Mental Disorders for Children and Adolescents (DISYPS-KJ) *[22], a battery of diagnostic checklists and symptom-specific questionnaires applying the criteria of the DSM-IV and ICD-10, thus allowing a standardized diagnosis of psychopathology. We used this inventory for these four diagnoses because we expected that these would be the most frequent diagnoses in the residential care setting [23]. The internal consistency of the DISYPS-KJ indicated by Cronbach's alpha is reported between .64 and .96 [22].

In addition to the aforementioned diagnoses-specific modules of the DISYPS-KJ, data about drug and alcohol abuse, tic-disorder, eating disorder, enuresis and encopresis were collected by interviewing the children and their caregivers. Clinical examination was performed by a trained psychologist. For a subsample of 13 adolescents, inter-rater agreement was determined by parallel examination of two independent investigators. Inter-rater reliability was found to be r = .93.

### Statistical analysis

Individual raw scores in the screening questionnaires were transformed into standard T-scores according to the German reference data. Means, standard deviations, and frequencies within the clinical range were calculated. Absolute frequencies of specific mental disorders were determined. Relative frequencies were determined by per cent relative to the total sample of 557 individuals participating with informed consent. Analyses of the children and adolescents who dropped out of the study after the screening revealed no significant differences compared with participants in the clinical examination in psychometric measures. Therefore the prevalence rates for the total study sample were estimated on the base of observed rates in the subsample participating in the clinical examination.

## Results

### Screening questionnaires

The analysis of the CBCL-scores of 132 children without informed consent showed that they did not differ in their global scores from those 557 participating with informed consent. Therefore we concluded that the study sample is representative for all children and adolescents in the participating institutions. From nine children neither the Youth Self Report (YSR) nor the Child Behavior Checklist (CBCL) could be evaluated, because both questionnaires filled out deficient or fragmentary.

The results of the screening questionnaires are demonstrated in table 2.

**Table 2:** Results of the screening with clinical questionnaires

Variables	Mean T-score	Standard deviation	% in the clincal range
CBCL-Int n = 667	60.1	10.1	55.5% > 59 T-points 18.3% > 69 T-points
CBCL-Ext n = 667	64.3	11.4	67.1% > 59 T-points 35.2% > 69 T-points
CBCL Total n = 667	64.4	9.8	72.1% > 59 T-points 33.4% > 69 T-points
YSR-Int n = 466	60.6	11.6	53.2% > 59 T-points 21.2% > 69 T-points
YSR-Ext n = 466	62.2	11.1	58.3% > 59 T-points 20.6% > 69 T-points
YSR-Total- n = 466	63.0	10.4	55.6% > 59 T-points 20.8% > 69 T-points

The mean CBCL total score was T = 64.4 with a standard deviation of 9.8. 33.4% of our residential care population reached CBCL total scores of at least two standard deviations above the mean in the normal population, and 70% of the whole study group reached CBCL total scores of at least one standard deviation above the normal. In the YSR, the children and adolescents reached a mean total score of 63.0 T-points (SD = 10.4). 55.6% scored one standard deviation and 20.8% scored two standard deviations above the mean of the German reference population.

452 individuals (81.2%) scored above the cut-off of 59 T-points in either the CBCL and/or the YSR, thus they fulfilled the criterion to enter clinical examination.

Table 3 presents the concordance of self-reported and caregiver-reported psychopathology. The results were convergent in 304 cases. In 94 cases the participants fulfilled the criterion because of the caregiver report. In 53 cases the results of the self-report of the children and adolescents led to a subsequent clinical examination. The correlation between YSR-total score and CBCL-total score amounts to *r *= .39.

**Table 3:** Concordance between self rating and rating of the residential care educator

Criterion T-Score > 59 n = 451	Rating of the educators CBCL < 60 T-points n = 134	Rating of the educators CBCL > 59 T points n = 317
Self Rating YSR < 60 T-points n = 175	81	94
Self Rating YSR > 59 T-points n = 276	53	223

In the CGAS 6.2% of the participants reached scores between 100 and 90 points, 17.5% between 90 and 80 points, 13.3% between 80 and 70, 16.2% between 70 and 60, 21.4% between 60 and 50, 13.3% between 50 and 40 points, 5.7% between 40 and 30 points, 4.8% between 30 and 20 points and 1.6% between 20–10 points.

### Clinical interviews

359 of the 452 children and adolescents with elevated CBCL and/or YSR scores were interviewed. 93 individuals dropped out of the study before the clinical examination could be performed. Most of them had left the residential care centre during the interval between screening and clinical examination, because they had finished their special school (n = 57). Others refused to participate in the interview (n = 26), and some adolescents could not be reached because they were in inpatient treatment (n = 7) or in a criminal youth custody unit (n = 3). The analysis of the screening data of these 93 individuals (73 male, 20 female) dropping out before the clinical examination showed that they were older (15.2 vs. 14.2 years in the mean) compared to the participants in the clinical interview, but they did not significantly differ in the three CBCL global scales (Total score (total), internalizing Score (INT), externalizing Score (EXT)).

According to the clinical interview, 88 participants (18.9%) did not fulfil the criteria of an ICD-10 diagnosis. 265 children and adolescents (57.1%) met the criteria of an ICD-10 diagnosis, 72 female (51.4%) and 193 male (59.6%) children and adolescents. The absolute frequencies of specific disorders and the relative frequencies related to the 557 participants of the study are demonstrated in table 4.

**Table 4:** Prevalence of mental disorders in the study group n = 464 drop-out n = 93 (73 male, 20 female individuals)

ICD-10 mental disorder*	Observed prevalence for 464 individuals frequency/%	Observed prevalence for the 140 female participants frequency/%	Observed prevalence for the 324 male participants frequency/%	Estimated** prevalence calculated for all 557 children and adolescents including drop-out
Inconspicuous in the screening	105 (22.6%)	31 (22.1%)	74 (22.8%)	18.9%
No mental disorder but conspicuous in the screening	88 (18.9%)	34 (24.3%)	54 (16.7%)	19.9%
Conduct disorder (F 91 + F 92)	115 (24.8%)	32 (22.9%)	83 (25.6%)	26%
ADHD with conduct disorder (F 90.1)	95 (20.5%)	9 (6.4%)	86 (26.5%)	22%
ADHD (F 90.0)	9 (1.9%)	1 (0.7%)	8 (2.5%)	2%
Depression and Dysthymia (F32 & FF34)	40 (8.6%)	18 (12.9%)	22 (6.8%)	10.4%
Anxiety disorders (F 4)	17 (3.7%)	10 (7.1%)	7 (2.2%)	4.0%
Eating Disorders F 5	2 (0.4%)	2 (1.4%)	0 (0.0%)	0.4%
Substance abuse (F 1)	39 (8.4%)	4 (2.9%)	35 (10.8%)	8.8%
Enuresis (F 98.0)	26 (5.6%)	8 (5.7%)	18 (5.5%)	6%
Encopresis (fF 98.1)	8 (1.7%)	1 (0.7%)	7 (2.2%)	1.8%
Tic-disorder (F 95)	8 (1.7%)	0 (0.0%)	8 (2.5%)	1.8%
mentally retarded (F70)	6 (1.3%)	3 (2.1%)	3 (0.9%)	1.4%
Any mental disorder	265 (57.1%)	72 (51.4%)	193 (59.6%)	59.9%

**Figure 2:**
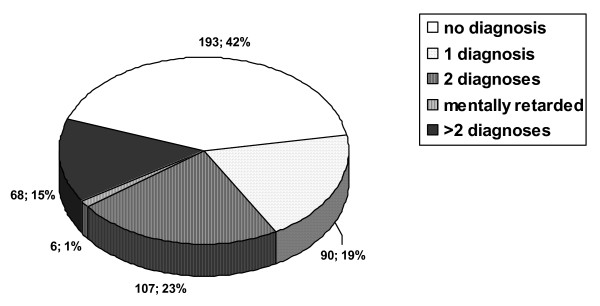
Observed comorbidity of mental disorders n = 464.

The most frequent diagnoses were conduct disorder (n = 115), combined ADHD and conduct disorder (n = 95), simple ADHD (n = 9), dysthymia/depression (n = 40), drug and alcohol abuse (n = 39), and enuresis nocturna (n = 26). The estimation of prevalence in the total sample, under the assumption of a similar frequency and of disorders in the 93 children and adolescents with positive screening results that dropped out of the study before clinical examination, is also demonstrated in table 4. Multiple diagnoses were frequent. 90 children and adolescents fulfilled the criteria for one diagnosis, 107 for two diagnoses and 68 for three or more diagnoses (see figure 2).

## Discussion

The aim of this survey study was to describe the prevalence of mental disorders of children and adolescents in German residential care institutions. In accordance with the results of survey studies of comparable populations from Great Britain or the United States [24-26,1], our study demonstrates a high amount of severely mentally disturbed children and adolescents. 59.9% of all children and adolescents fulfilled the criteria for an ICD-10 diagnosis, 81.15% reached a CBCL or YSR global score in the clinical range, about one third of the study sample scored two standard deviations or more above the mean of the normal population.

The high prevalence of conduct disorders and combined ADHD with conduct disorder and the extremely high externalizing CBCL-scores indicate that disruptive behavior is the main problem in residential care institutions. It is known that male adolescents have a higher prevalence of externalizing disorders, compared to female peers. On the other hand, more female adolescents suffer from internalizing disorders. This trend is supported by the findings of our study, and the over-representation of males contributes consequently to the predominance or externalizing disorders in our study group. With regard to the known poor prognosis of externalizing disorders, including the risk of developing antisocial personality disorders and/or drug addiction [27,28], our results indicate a severe burden for the residential care institutions.

The high rate of 37% comorbid disorders and the significant impairment of psychosocial functioning as demonstrated by the CGAS with about nearly 50% in a handicapped range support the impression of a predominance of severe disorders in this population. This is also a matter of costs in the health system because adolescents with comorbidity of depression and conduct disorders generate in the long run higher costs for using mental and social services than children and adolescents without comorbidity [29].

One part of our sample suffers from undetected mental problems, whereas most of the mentally disturbed children and adolescents in our study group have persistent disorders and had already been in contact with the mental health system. But only a few of them were in current treatment at the time of our study. Blower et al. [26] reported similar observations in their sample and postulated that one problem for current treatment is waiting and travel times for the residential care stuff.

In summary, our study adds additional evidence (from an European perspective) that children and adolescents in youth welfare and especially in group homes are at high risk for the development of mental disorders. Children out of residential care are more vulnerable for mental disorders because a lot of biological and psychosocial risk factors are concentrated among this group.

Some limitations of this study have to be mentioned. The sensitivity of our clinical assessment for a comprehensive scope of mental disorders in childhood and adolescence was limited. Because of limited financial resources, it was necessary to compromise and use checklists and questionnaires. For the same reason, psychometric tests of cognitive ability, learning disabilities or other developmental disorders were not included in our assessment. In consequence developmentally retarded children could not be identified with sufficient reliability. By using the DISYPS-KJ diagnostic checklists, instead of another more time consuming standardized interview, the most common disorders could be diagnosed with sufficient reliability.

Due to the non-comprehensive scope of our standardized clinical assessment, our results represent rather an underestimation of the real prevalence of mental disorders in the study sample. The real prevalence in our study group might be higher, because our method was not sufficiently sensitive for several relevant clinical diagnoses such as pervasive developmental disorders, PTSD, attachment disorders, and mental or developmental retardation. Especially PTSD and other trauma related disorders might be common in this high risk population, but one demand of the ethic committee was to avoid re-traumatization. Trauma related problems could not be accessed in a ethical correct way and non time consuming way by using diagnostic checklists.

On the other hand, some strengths of our methodology support the value of our findings. The two-step and multi-informant design allowed a control of our diagnostic procedures and cross-validated the results regarding psychopathology. The relatively large sample size of 689 children, representing nearly one per cent of the total German residential care population, minimizes the chance of a relevant selection bias.

## Conclusion

Consequences of our findings have to be discussed with regard to the mental health care needs of this high-risk population. As it is more likely for a child or adolescent in residential care to suffer from a mental disorder than to be healthy, monitoring mental health already at admission to child and youth welfare system will be necessary. There is a need for psychiatric liaison-services within the child welfare system in order to provide sufficient diagnostic and therapeutic services. Professionals within the child welfare system should be trained in caring for mentally disturbed children and adolescents. Co-operation between child and adolescent psychiatrists, psychotherapists, social workers and caregivers within the residential care institutions should strengthen the chance of continuous care and avoid repeated breaking-offs. Therapeutic options in co-operation between residential care institutions and child and adolescent psychiatry should be taken including appropriate diagnostic procedures, continued psychotherapy, staff counseling and medication. There is a need for delivering effective interventions for these children and adolescents with often multiple mental disorders in the residential care institutions. Therefore it would be important to create further therapeutic opportunities in co-operation between residential care institutions and child and adolescent psychiatry in order to avoid unnecessary admissions to psychiatric wards. A rapprochement of the professions and institutions might be able to reduce the reluctance and fear of stigmatization of young people in residential care institutions to become involved with the child and adolescent psychiatric services.

Epidemiological surveys in most countries usually are family based. Our findings and the results of other studies on children in institutional care show that this leads to an underestimation of the general prevalence and severity of psychiatric disorders. This error varies with the proportion of institutionalized children in a country. For future epidemiological studies or normative samples other sampling procedures than family based should be carried out. For some clinical studies we need an oversampling of risks and well defined high-risk populations. Children in institutions accumulate social and biological risk factors and show a much higher frequency of psychiatric disorders in comparison to the population living in their natural families. With respect to future health costs more intervention studies should be carried out in this high risk population suffering from co-morbidities and a high number of psychosocial risks.

## Authors' contributions

MS conceived the design of the study, performed data analysis and drafted the manuscript. LG was leader of the study. He designed the study and advised the statistical analysis. JN participated in the design of the study and supported the data collection. JMF was doctoral advisor and raised the third party funds to realize the study and advised study design, analysis and interpretation of results. All authors read and approved the final manuscript.
